# Endovascular stroke treatment using balloon guide catheters may reduce penumbral tissue damage and improve long-term outcome

**DOI:** 10.1007/s00330-020-07260-3

**Published:** 2020-10-10

**Authors:** Maria T. Berndt, Mayank Goyal, Marios Psychogios, Johannes Kaesmacher, Tobias Boeckh-Behrens, Silke Wunderlich, Claus Zimmer, Benjamin Friedrich, Christian Maegerlein

**Affiliations:** 1 Department of Diagnostic and Interventional Neuroradiology, Klinikum rechts der Isar, School of Medicine, Technical University of Munich, Munich, Germany; 2 Department of Radiology, Department of Clinical Neurosciences, University of Calgary, Calgary, Canada; 3 Department of Neuroradiology, University Hospital Basel, Basel, Switzerland; 4 Department of Neuroradiology, Inselspital, University Hospital of Bern, University of Bern, Bern, Switzerland; 5 Department of Neurology, Klinikum rechts der Isar, School of Medicine, Technical University of Munich, Munich, Germany

**Keywords:** Stroke, Reperfusion, Angiography, Diffusion tensor imaging

## Abstract

**Objectives:**

During mechanical recanalization of large vessel occlusions (LVO), the use of proximal flow arrest with balloon guide catheters (BGC) was shown to be associated with better angiographic and even clinical outcome. The aim of the study was to analyze the impact of BGC use on microstructural alterations in the salvaged penumbra.

**Methods:**

All patients who underwent mechanical recanalization of LVO of the anterior circulation were reviewed within a prospective stroke registry of a single comprehensive stroke center. Fifty-two patients received an admission CT perfusion together with post-interventional diffusion tensor imaging. Technical details such as BGC usage were correlated with microstructural integrity changes of the salvaged gray matter through the mean diffusivity (MD) index. Moderation analysis was performed to test the interaction of BGC on the correlation between angiographic and clinical outcomes.

**Results:**

For all patients with complete reperfusion, microstructural integrity changes with lowered MD index were found within the salvaged penumbra for cases of non-BGC usage (mean − 0.02) compared to cases with BGC usage (0.01, *p* = 0.04). The importance of complete reperfusion for good clinical outcome is predominantly based on patients treated with BGC (effect 2.78, *p* = 0.01 vs. for non-BGC: 0.3, *p* = 0.71).

**Conclusions:**

The lowered MD index early after mechanical recanalization without BGC usage can be interpreted as microstructural ischemic damage of the salvaged penumbra. It was shown that achieving complete reperfusion in a setting of BGC usage with proximal flow arrest minimizes penumbral damage and improves long-term outcomes.

**Key Points:**

*• Microstructural ischemic damage can be reduced by using proximal flow arrest during endovascular treatment with balloon guide catheter*.

*• Complete reperfusion in a setting of balloon guide catheter minimizes penumbral damage and improves long-term outcome*.

**Electronic supplementary material:**

The online version of this article (10.1007/s00330-020-07260-3) contains supplementary material, which is available to authorized users.

## Introduction

Endovascular mechanical thrombectomy (MT) is used as the current standard procedure for large intracranial vessel occlusions (LVO) of the anterior circulation, based on the major five randomized trials, in which so-called stent retrievers were an integral part of endovascular therapy (EVT) [1–5]. Recently, the indication has been extended beyond the studies mentioned above to later time windows and smaller vessels [6–8]. A further important focus is set on technical aspects, especially on identifying adjunctive techniques and combination techniques to improve the technical procedure itself with the aim to increase the rate of complete reperfusions and, as a result of this, improve the patients’ outcome.

Especially the use of balloon guide catheters (BGC) has been established within the standard procedure in many neuro-interventional institutions by now, as it became evident that improved angiographic and clinical results can be achieved by using this technique [9–13]. This is undoubtedly affected by a reduction of distal (micro)emboli [14]. The advantage when using BGC is the establishment of proximal flow arrest or even flow reversal during the thrombectomy procedure, whereas the antegrade bloodflow is upheld when using normal guiding catheters or long sheath. The proximal flow control allows to remove small thrombus fragments or debris that might otherwise go downstream to more distal, smaller vessels, leading to new and often irreversible ischemia. These emboli often cannot be removed because they are located too far peripherally and/or (in the case of microemboli) are not recognizable at all.

The aim of acute stroke treatment is to save the so-called penumbra. “Penumbra” means the potentially salvageable brain tissue that is at risk of definite infarction if no successful reperfusion therapy will be performed immediately.

The penumbra presents the range of cerebral perfusion between temporary loss of electrical activity and irreversible neuronal depolarization and plays an essential role as the degree of “tissue at risk” can be assessed [15, 16]. This salvageable brain parenchyma can be estimated by perfusion CT or MRI and therefore became an important selection marker for EVT [6, 7, 17]. The penumbra is a region suffering from a temporary lack of supply in oxygen and nutrients that potentially results in ischemic tissue damage, dependent on the ischemic time and collateralization [18].

Previous analyses have suggested that microstructural alterations also occur in the salvaged penumbra, which does not undergo final infarction [19–21]. Microstructural analysis can be done by use of diffusion tensor imaging (DTI) analysis. Here, the mean diffusivity (MD) index of the penumbral gray matter that measures cortical MD asymmetry compared with the contralateral, non-affected hemisphere is reduced in case of penumbral damage after endovascular treatment [22]. Associations of the penumbral damage measured by the MD index with angiographic results and clinical outcomes exist that warrants the evaluation of strategies to protect the penumbral tissue integrity [22].

We hypothesize that the processes that take place in the penumbra until successful reperfusion are influenced by technical specifications of the MT technique such as usage of BGC.

Therefore, the primary aim of the present study was to analyze the impact of applied procedural techniques on penumbral processes. A specific focus was laid on the use of BGC, and the microstructural changes within the salvaged penumbra in the acute stroke phase after mechanical recanalization of an acute intracranial vessel occlusion were assessed. Additionally, the interaction of the techniques on the correlation between angiographic and clinical outcomes should be tested.

## Material and methods

### Sample and patient description

The study cohort is based on a prospectively collected stroke registry of a single comprehensive stroke center. All patients treated with MT between April 2016 and December 2018 were included. The database query revealed four hundred thirty-nine patients that had undergone EVT due to LVO of the anterior circulation (middle cerebral artery or carotid-T, cohort already described in [22]).

Inclusion criterion was a quality-sufficient MRI acquisition in the acute post-stroke phase (median 3 days, IQR 3–4 days, maximum 7 days) after MT, including diffusion tensor imaging (DTI) and structural T1-weighted imaging (*n* = 192). Exclusion criteria were incomplete or insufficient MRI acquisition (*n* = 20), space-consuming malignant infarction, or intracranial hemorrhage (*n* = 7). From the residual 165 patients, 52 met the further required inclusion criteria: (a) in-house-acquired admission CT perfusion imaging that was essential to determine the penumbral tissue immediately before EVT; (b) clearly identifiable angiographic technique (no mixed techniques, e.g., change of technique within one intervention, or only ADAPT; for details, see below section “Angiographic data and differentiation of interventional techniques”).

For the final study cohort, the prospectively collected clinical and imaging data were extracted from the registry. Certified neurologists assessed the National Institutes of Health Stroke Scale (NIHSS) score at the time of admission and at the time of MRI acquisition. The modified Rankin Scale (mRS) score was used to measure disability at day 90 after stroke onset, either on a routinely scheduled clinical visit or by a structured telephone interview.

This study was approved by the local ethics committee and the need for patient consent was waived.

### Angiographic data and differentiation of interventional techniques

For all study patients, the procedure of MT was classified by two experienced neuro-interventionalists (C.M., B.F.) in consensus regarding the technical approach. The following categories of intervention techniques were differentiated (see also supplementary material, section “Technique of endovascular thrombectomy”):

The BGC group (*n* = 29) is composed of all patients that were treated with proximal flow arrest in a setting of BGC usage (PROTECT or PROTECT^PLUS^):

- PROTECT: PRoximal balloon Occlusion TogEther with direCt Thrombus aspiration during stent retriever thrombectomy (*n* = 11) [23]: BGC + aspiration catheter + stent retriever (complete retrieval of the stent retriever into the aspiration catheter)
- PROTECT^PLUS^ (*n* = 18) [12]: BGC + aspiration catheter + stent retriever (only partial retrieval of the stent retriever into the aspiration catheter and withdrawal of both as a unit into the BGC).

The non-BGC group (*n* = 23) is composed of all patients treated without BGC usage:

- SOLUMBRA [24]: Guiding catheter + aspiration catheter + stent retriever (the stent retriever is being retracted into an aspiration catheter at the occlusion site).

The interventionalists were free to decide which technique to use. The decision to use BGC was mainly driven by increasingly published studies that pointed to a technical and possible clinical advantage. Anatomical peculiarities like coiling or kinking of the ICA were no exclusion for BGC as the use of long 8-F/9-F sheaths enables sufficient stability for using BGC.

Due to the exclusion criteria of mixed techniques (e.g., change of technique within one intervention), patients treated with only ADAPT ([25], guiding catheter + aspiration catheter without stent retriever application (*n* = 13)) were excluded for direct comparisons of BGC vs. non-BGC. A possible bias within this group arises from the high conversion rates of this approach (secondary stent retriever application). Consequently, procedural and outcome results of the cases that used only the ADAPT technique are artificially good.

By using this classification, all patients within the groups of BGC and non-BGC were treated straight by intracranial stent retriever application that makes them comparable and avoids bias. For none of the patients within the study cohort, procedural complications occurred or permanent stent implantation (extra- or intracranial) was necessary.

The modified thrombolysis in cerebral infarction (mTICI) score [26] was determined by two experienced neuro-interventionalists (C.M., B.F.) in consensus. Complete reperfusion was defined as mTICI 3. Time of the groin puncture, time of the reperfusion, and corresponding procedure times were taken from the existing database. The difference between the time of groin puncture and reperfusion is defined as reperfusion time in the following. If no successful reperfusion could be achieved (mTICI < 2b), the last digital subtraction angiography (DSA) run was defined as the time endpoint.

### Assessment of the microstructural integrity of the salvaged penumbra

**Fig. 1:**
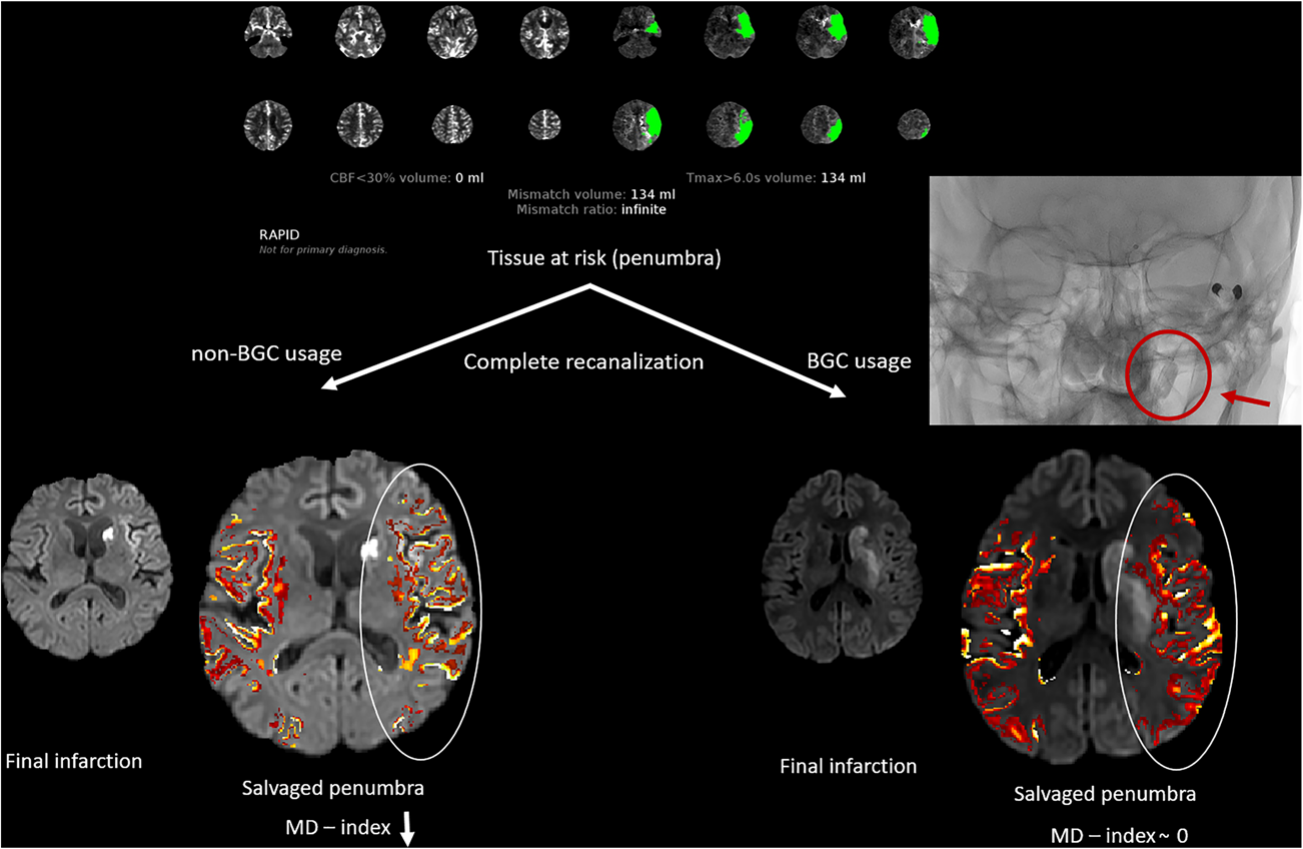
Examples of assessing microstructural integrity of the salvaged penumbra (representative example of RAPID evaluation in CT perfusion imaging). After complete recanalization of an acute occlusion of the middle cerebral artery, MD values of the affected side are lowered in comparison to the contralateral side only for the case of non-BGC usage. BGC, balloon guide catheter

Salvaged penumbral tissue was identified for each patient. Final infarct assessed with an MRI acquisition including DTI in the acute post-stroke phase was subtracted from the tissue at risk (penumbra) of co-aligned admission CT perfusion imaging. Hereby, salvaged penumbra was identified in the acute post-stroke phase and its microstructural integrity was analyzed (see Fig. 1 and supplementary material).

#### Penumbra in admission CT imaging

To identify the tissue at risk (penumbra) in the acute stage immediately before mechanical recanalization, CT perfusion imaging was acquired and post-processed by the fully automated software RAPID (iSchemaView Inc.). Perfusion maps were verified by an at least 3-year experienced neuroradiologist (M.B.) (further details can be found in the supplementary material).

#### MRI data acquisition and assessment of final infarction

The area of final infarction was identified by the use of DTI in the acute post-stroke phase (for technical details, see supplementary material). Infarction volume was assessed by using semi-automatic segmentation software (ITK-SNAP, www.itksnap.org [27]) with subsequent quantitative analysis. Punctuate DWI lesions distant to the infarction core within the brain regions supplied by the treated vessel were classified as peripheral emboli and were also included in the final infarction assessment.

#### Identification and microstructural analyses of the salvaged penumbra

By co-registering of admission CT perfusion imaging and MRI acquisition in the acute post-stroke phase, salvaged penumbra was calculated for each patient by a subtraction of tissue at risk and final infarction. After processing of DTI images using FSL’s FDT toolbox, MD maps were created. To find gray matter alterations within the salvaged penumbra, MD values of the penumbra on the infarcted side (I) were assessed and compared with MD values of the corresponding voxels on the contralateral, non-affected side (H). MD index was calculated by using the following formula: MD index = (MD_I_ − MD_H_) / (MD_I_ + MD_H_). A negative MD index means lower MD values within the penumbral gray matter compared with the non-affected side. In a previous work, this MD index was effectively used for characterizing microstructural integrity within the salvaged penumbra [22]. For the comparison of MD index between the different techniques, the precondition of mTICI 3 should be met, because incomplete reperfusion would impact penumbral tissue integrity.

Technical details of the co-registration process, DTI processing, and quantitative MD analyses for calculation of MD index can be found in the supplementary material.

### Statistical analysis

Mean value comparison of MD index or volume of infarction was performed by means of a two-sample *t* test for independent samples. Pearson’s correlation was used to find a possible coherence between MD index and volume of infarction. Chi-squared statistic was applied to test a difference in the occurrence of peripheral emboli for BGC/non-BGC groups.

Logistic regression analysis was performed to test for an association between complete reperfusion (mTICI 3) and good clinical outcome (mRS ≤ 2 after 90 days) under consideration of the covariates age, sex, and reperfusion time. Moderation analysis was performed to check the interaction of the subgroups of BGC/non-BGC on the association between complete reperfusion and good clinical outcome.

## Results

### Patient characteristics

In total, 52 patients met the inclusion criteria as described above. An overview of demographic, clinical, and interventional data of patients and a comparison between the BGC and non-BGC groups can be found in Table 1.

**Table 1:** Basic demographic, clinical, and interventional data as well as volume of infarction, incidence of peripheral emboli, and MD index of the non-BGC and BGC groups, subdivided into PROTECT and PROTECT^PLUS^ (see manuscript for technical explanations). Results of group comparisons between non-BGC and BGC groups: “*” indicates statistically significant results on the 5% level (Mann-Whitney *U* test or chi-squared statistic). *mTICI*, modified thrombolysis in cerebral infarction; *NIHSS*, National Institutes of Health Stroke Scale; *mRS*, modified Rankin Scale

Characteristic	Non-BGC (*n* = 23)	BGC (PROTECT (*n* = 11)/PROTECT^PLUS^ (*n* = 18))	Group comparison (non-BGC vs. BGC), *p* value
Age (year), median, IQR	80 (73–88)	69 (60–77)/ 69 (62–77)	0.03*
Sex, *n* (male/female)	10/13	5/6 10/8	> 0.05
Interventional parameters, time (median, IQR)			
Groin puncture to recanalization (min)	27 (23–37)	38 (24–53)/ 27.5 (20–40)	> 0.05
Symptom onset to recanalization (min)	193 (144–268)	281 (200–417)/ 158 (136–206)	> 0.05
Number of maneuvers	2 (1–3)	2 (1–4)/ 2 (1–2)	> 0.05
mTICI score (*n*)			> 0.05
0–1	1	1/0	
2a	0	1/0	
2b	8	4/6	
3	14	5/12	
Additional intravenous thrombolysis (*n*)	11	4/7	> 0.05
NIHSS (median, IQR)			
Pre-treatment	10 (6–16)	13 (6–20)/ 10 (6–14)	> 0.05
Time of MRI acquisition	1 (0–5)	2 (0–10)/ 1 (1–3)	> 0.05
Time of discharge	1 (0–3)	3 (0–10)/ 2 (0–4)	> 0.05
mRS score (after 90 days, *n* = 52)			> 0.05
0–2 (63%)	11	5/9	
> 2 (37%)	8	5/4	
Time between recanalization and MRI acquisition (days, median, IQR)	3 (3–5)	3 (3–4)/ 3 (3–4)	> 0.05
Volume of infarction (ml, mean ± SD)	23.3 ± 44.6	45.2 ± 76.2/ 24.9 ± 30.4	> 0.05
Incidence of peripheral emboli	12/13 (92%)	7/14 (50%)	0.02*
MD index (mean ± SD)	− 0.019 ± 0.047	− 0.012 ± 0.040/ − 0.005 ± 0.048	0.04* (precondition: mTICI 3)

### Impact of BGC on macro- and microstructural changes within the salvaged penumbra

For completely reperfused patients, there is a significantly lower incidence of peripheral emboli in the BGC group (50%) in comparison with the non-BGC group (92%, *p* = 0.016).

**Fig. 2:**
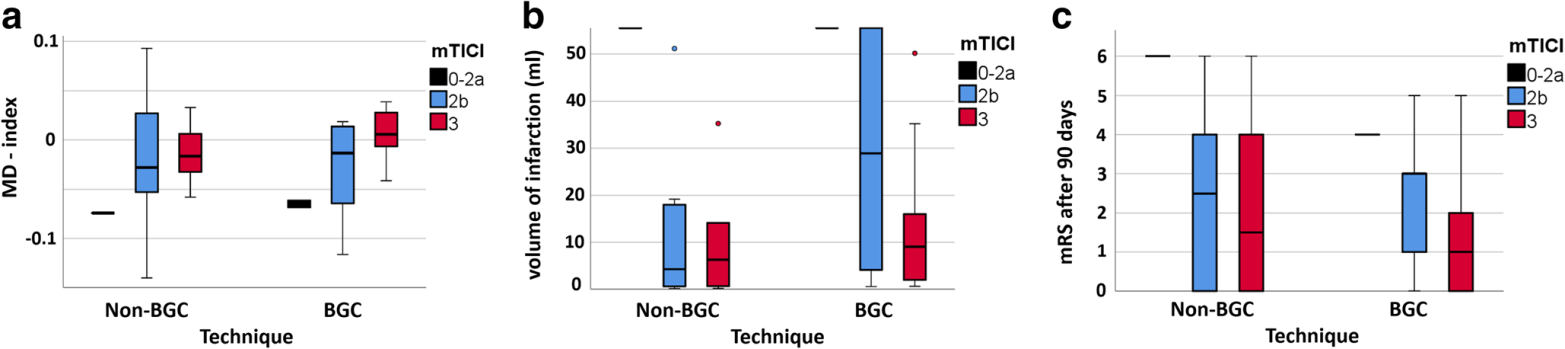
Distributions of MD index (**a**), volume of infarction (**b**), and mRS (**c**) after 90 days for the technical subgroups BGC/non-BGC in dependency of reperfusion success (measured by mTICI). BGC, balloon guide catheter; mTICI, modified thrombolysis in cerebral infarction

The distribution of MD index within the gray matter of the salvaged penumbra for the group of BGC and non-BGC is shown in Fig. 2a. Values of MD index are mainly negative. For both groups, MD indices rise with the better angiographic outcome, measured by mTICI.

For directly comparing penumbral integrity of the different techniques, the precondition of mTICI 3 should be met. MD index of the BGC group shows significantly higher values (mean ± SD: 0.009 ± 0.035) than MD index of the non-BGC group (mean ± SD: − 0.015 ± 0.027, *p* = 0.04).

Values of MD index are inversely correlated to the volume of infarction (Pearson’s *r* = − 0.32, *p* = 0.01). To exclude an influence of the volume of infarction on the impact of BGC on MD index as mentioned above, volume distributions for the technique groups in dependency of angiographic success can be found in Fig. 2b. The volume of infarction is reduced with a better angiographic outcome for both groups. No volume differences can be found between the BGC (mean ± SD: 32.6 ± 52.3 ml) and non-BGC (23.3 ± 44.6 ml, *p* = 0.5) groups.

### Impact of BGC on angiographic and clinical outcome

Complete reperfusion success (mTICI 3) is significantly associated with good clinical outcome (mRS ≤ 2 after 90 days) in a logistic regression analysis (odds ratio 7.00, *p* = 0.017). The covariates age, sex, and reperfusion time showed no significant influence on the analysis.

In a second step, the impact of the subgroups BGC/non-BGC on this association was tested in a moderation analysis under consideration of the abovementioned covariates. The conditional effect of complete reperfusion success on good clinical outcome remained significant for the BGC group (effect 3.29, 95% CI 0.80 to 5.77, *p* = 0.01). For the non-BGC group, the conditional effect was lowered and lost its significance on the 5% level (effect 1.5, 95% CI − 0.42 to 3.46, *p* = 0.12). That implies that the importance of mTICI 3 reperfusion for good clinical outcome is predominantly based on patients treated with BGC.

Distribution of mRS values for the technique groups in dependency of angiographic success can be found in Fig. 2c.

## Discussion

The present study shows that macroscopic salvaged penumbra after MT of an LVO is microstructurally affected in dependency of the applied interventional technique. The patients that had undergone complete reperfusions using BGC showed no relevant discrepancy of MD values within the salvaged penumbra compared to the contralateral side. Patients who were treated without BGC usage presented negative penumbral MD indices. These negative values indicate microstructural ischemic damage of the salvaged penumbra that was shown to impact on clinical outcome [22].

The importance of BGC use for the recanalization process was additionally shown in the following context: The clinical relevance of achieving complete reperfusion was discussed in the past [28]. By performing subgroup analyses in the present study, the importance of mTICI 3 reperfusion for good clinical outcome is predominantly based on patients treated with BGC. That is in line with the structural results of preserved microstructural tissue integrity in patients with angiographic mTICI 3 results only when using BGC.

These findings reinforce the necessity of achieving complete reperfusion in a setting of BGC usage during MT. With this, the proximal flow arrest is utilized for minimizing microstructural tissue damage in the salvaged brain.

Considering the history and current developments of mechanical thrombectomy, their impact on the integrity of the saved tissue has not been investigated and thus was the primary aim of the present study. All patients were treated in a single comprehensive stroke center and due to the long-term data acquisition between 2016 and 2018, different procedural techniques were applied for mechanical recanalization. This enables a comparison between these techniques regarding structural consequences within the penumbra. Especially the beginning of BGC usage constitutes an innovation with special improvement of the intraprocedural setup.

Previous studies already worked out the main benefits of the BGC application utilizing its proximal flow arrest capacity that results in improved angiographic and clinical outcomes [9–11, 13]. The physiological mechanisms were investigated in in vitro studies and showed an increased suction effectiveness with the consequences of easier clot removal, shorter procedure times, and reduction of distal emboli [14, 29].

Correspondingly, the present study showed a significant lower incidence of peripheral emboli (macroscopic damage) by BGC usage. The main new finding is the preserved microstructural integrity (higher MD index) when using BGC that is known to impact the clinical course of patients [22]. To avoid a bias induced by the well-known significantly higher rates of complete reperfusion for BGC usage [11], the precondition of complete reperfusion success was set for the comparison analysis.

As expected, there is an interaction of MD index of the salvaged penumbra with the volume of infarction. The more the ischemic demarcation is visible, the higher the integrity loss of salvaged penumbra can be measured. This coherence makes it necessary to check the distribution of ischemic volume for the technical subgroups (BGC/non-BGC) that in the end showed no volume differences. That confirms the supposed hypothesis that BGC usage saves the microstructural integrity of the salvaged penumbral tissue for the case of complete reperfusion, independently from the extent of final infarction. However, this point does not apply to the same extent to incomplete reperfusions. The remaining vessel occlusions impact penumbral tissue integrity so much (broad range of infarction volume for both groups) that no clear microstructural benefit is detectable for the cases of BGC usage, but only a trend to higher MD indices.

It can be supposed that clot fragmentation and micro-embolization into the occlusion-dependent brain tissue during MT would affect microstructural, ischemic-like damage within the salvaged penumbra. This damage can be measured by MD index that gives information about the tissue integrity of the salvaged gray matter, compared with the contralateral non-affected hemisphere. By assessing MD values using DTI, changes of the molecular diffusion rate can be detected that give information about subtle integrity alterations [30]. For the present study, MD values were investigated, because the focus was set on the gray matter integrity that reflects the ischemic processes directly.

A previous study showed that MD index for gray matter is a very accurate parameter for characterizing penumbral integrity [22]. Certainly, there are additional imaging criteria that may characterize penumbral microstructural changes that were not in the focus of the present study: White matter integrity is less prone to get infarcted and depends on the affected gray matter in respect of processes like early disintegration. Therefore, analysis of the white matter was waived in the present study. That could be considered in future studies, as white matter alterations could give important information about secondary phenomena (e.g., degeneration) in the long-term follow-up.

Finally, it cannot be proven that the microstructural damage in the salvaged penumbra, that was shown for the non-BGC group, did not exist before recanalization. However, the mTICI 3/BGC group showed no microstructural damage, which implies that this developed secondarily, possibly induced by clot fragmentation and micro-embolization. A bias in performing the groups of BGC/non-BGC cannot be excluded, but it is rather unlikely in the study’s cohort: since the implementation of BGC in this single comprehensive stroke center, nearly every endovascular treatment was made by the use of BGC, and the non-BGC group consists of patients mostly treated in 2016–2017, before BGC was routinely applied. The exclusion of ADAPT cases reduces the generalizability of the findings as these are only applicable for the case of stent retriever application. A further limitation is the moderate sample size within the groups that makes a fine matched analysis too powerless. Especially in the beginning phase of the study, not all patients who were mechanically recanalized were examined with MRI, arising from internal organization problems (lack of MRI capacity, transfer of patients back to the referring hospitals, etc.). Patients with disability for an MRI examination could also not be included. These patients often present extended ischemia or space-consuming hemorrhage that makes a DTI analysis impossible. This subgroup of patients with distinct worse outcome is missing in the analysis.

## Summary/conclusions

In summary, the present study highlights the importance of technical aspects during mechanical recanalization of acute LVO. With the use of DTI, it was shown that achieving complete reperfusion in a setting of BGC usage minimizes microstructural tissue damage in the salvaged penumbra and improves long-term outcome. This reinforces the importance of using BGC in endovascular stroke treatment.

## Electronic supplementary material

ESM 1 (DOCX 32 kb)

## Abbreviations

BGC: Balloon guide catheter
CT: Computed tomography
DTI: Diffusion tensor imaging
EVT: Endovascular therapy
MD index: Mean diffusivity index
MRI: Magnetic resonance imaging
mRS: Modified Rankin Scale
MT: Mechanical thrombectomy
mTICI: Modified thrombolysis in cerebral infarction
NIHSS: National Institutes of Health Stroke Scale
non-BGC: Usage of normal guiding catheters instead of BGC
