# Supplementary material for: Endovascular stroke treatment using balloon guide catheters may reduce penumbral tissue damage and improve long-term outcome
Source: Eur Radiol. 2020 Oct 10;31(4):2191–8. doi: 10.1007/s00330-020-07260-3 (PMC7979594; doi:10.1007/s00330-020-07260-3)
Supplement: Supplementary file 1 — (DOCX 32 kb) [file 330_2020_7260_MOESM1_ESM.docx]

**Endovascular stroke treatment using balloon guide catheters may reduce penumbral tissue damage and improve long-term outcome.**

**Supplementary material, referring to Material and Methods**

**Angiographic data and differentiation of interventional techniques**

**Technique of endovascular thrombectomy**

All interventions were performed via a femoral artery access. In the case of BGC usage a 9F Cello (ev3) or 8F FlowGate2, (Stryker) was introduced into the ipsilateral ICA. In the case of non-BGC usage an 8F guide catheter (Vista bright tip, Cordis/ NeuronMAX 088, Penumbra) was used, respectively.

For all maneuvers, a large bore aspiration catheter was introduced into the distal ICA or MCA (ACE 68, Penumbra; SOFIA, MicroVention; or Catalyst 6, Stryker). The following Stent retrievers were used: pREset, Phenox; TrevoXP ProVue, Stryker; Solitaire 2, Medtronic. Therefore, the device was introduced via a microcatheter (Trevo 18, Stryker) and deployed. The microcatheter was carefully removed afterward to increase aspiration applied by an electrical pump (Pump MAX, Penumbra) during the stent retriever retractions. Additional manual aspiration with a large syringe on either the guiding or BGC was performed simultaneously.

**Assessment of the microstructural integrity of the salvaged penumbra**

**Penumbra in admission CT imaging**

*CT Data Acquisition*

Standard non-helical cerebral computed tomography was performed on a 64-row CT scanner equipped with a 40-mm detector (Philips Brilliance 64, Philips Medical Systems B.V.). Beside non-contrast images and computed tomography angiography, a perfusion image was acquired by using the following parameters: 120 kV, 400 mAs, with 5mm section thickness. A dual-head power injector with an 18-G i.v. access was used for contrast injection. A delay of 5 seconds was applied after injecting 40ml Imeron (400 mg I/mL Imeron 350, Bracco Imaging) at a flow rate of 6ml/s, followed by 90 mL NaCl.

*Penumbra assessment in CT perfusion using RAPID*

For postprocessing of the acquired CT perfusion images, RAPID (iSchemaView Inc) was used. This software is an operator independent, fully automated image processing and visualization tool and allows online estimation of perfusion maps and mismatch masks [1]. For assessing penumbral tissue, the individual hypoperfusion mask was extracted and hypo-perfused tissue was estimated at thresholded values of Tmax delay >6 seconds [1-3].

**MRI Data Acquisition and assessment of final infarction**

MRI data were acquired on a 3T Philips scanner (Achieva, Philips Medical System) with standard eight-channel head coils using consistent sequences and parameter settings.

Diffusion tensor images were acquired using a single-shot spin-echo echo-planar imaging sequence, resulting in one non-diffusion weighted image (b = 0 s/mm2) and 15 diffusion weighted images (b = 800 s/mm2, 15 non-colinear gradient directions) covering whole brain with following parameters: echo time (TE) = 55 ms, ﬂip angle = 90°, ﬁeld of view (mm) = 224 x 224 x146, 73 transverse slices, slice thickness = 2 mm, and 0 mm interslice gap, voxel size (mm) = 2 x 2 x 2.

A whole-head, high resolution 3D gradient echo T1-weighted image was acquired using the following parameters: echo time (TE) = 4 ms, repetition time (TR) = 9 ms, ﬂip angle = 8°, ﬁeld of view (mm) = 240 x 252 x 200.25, 267 sagittal slices, slice thickness = 1 mm, and 0 mm interslice gap, voxel size (mm) = 1 x 1 x 1.

Infarction volume was assessed from DTI data within the acute post-stroke phase. Area of infarction was segmented by using semiautomatic segmentation software (ITK-SNAP, www.itksnap.org, [4]) with subsequent quantitative analysis. On consideration of the reconstructed trace and ADC-maps, an at least three-years experienced neuroradiologist (M.B.) acquired the entire infarcted lesion by thresholding and manual adaptation for each patient. Subsequently, lesion volumes were extracted.

**Identification and microstructural analyses of the salvaged penumbra**

*Diffusion Tensor Imaging (DTI) and 3D T1 Data Processing*

Diffusion data were processed using FSL's FDT toolbox. Eddy-current distortion and head motion were corrected by linear registration of all diffusion-weighted images to the first b0-volume. Brain-tissue extraction was performed by removing the skull and non-brain tissue using FSL BET [5]. The tensor model was fitted with DTIFIT (FSL) to obtain images of mean diffusivity (MD).

FAST (FMRIB's Automated Segmentation Tool) was applied to segment the native 3D T1 images into different tissue types (grey and white matter, cerebrospinal fluid) and to correct for spatial intensity variations [6].

Each image was visually checked by an at least three-years experienced neuroradiologist (M.B.) to identify data corrupted by artifacts.

*Coregistration of CT and MRI imaging and quantitative MD analyses*

After using FSL BET for brain extraction of the anatomical CT perfusion images [5], a two-step transformation procedure was applied to register the individual CT image to the diffusion image. In the first step, linear (affine) transformation was performed between CT image and brain extracted and bias-corrected native T1 image of the same patient using FLIRT (FMRIB's Linear Image Registration Tool, [7; 8]) with twelve degrees of freedom and correlation ratio cost function. In the second step, the same procedure was applied to register the brain extracted b0-image (diffusion space) of each subject to corresponding native T1 image. These individual diffusion-to-native and native-to-CT linear transformation matrices were combined that resulted in individual diffusion-to-CT transformations and their corresponding inverses. These warp-fields were then applied to the individual hypoperfusion mask in CT space, extracted from CT perfusion images by the use of RAPID (see section ´Penumbra assessment in CT perfusion using RAPID´ above), to align them into each subject’s individual diffusion space with the implementation of nearest neighbor interpolation, followed by a visual check by a three-years experienced neuroradiologist (M.B.). In the intermediate step of T1 space, information of FAST segmentation was implemented to identify and map only the grey matter of the hypoperfusion masks. Prior segmented ischemic infarction maps (see section ´ MRI Data Acquisition and assessment of final infarction´) were then subtracted from the hypoperfusion mask to get a mask (penumbral mask), that contains grey matter of the salvaged penumbral tissue, that in the end did not show visible infarction. MD-values of this penumbral mask were extracted and averaged using Matlab-based in-house software.

To find intraindividual grey matter alterations, MD-values of the corresponding voxels in the contralateral, healthy, non-affected side (H) were assessed and compared with MD-values of the infarcted side (I). MD-index was calculated by using the following formula: MD-index = (MD_I_ – MD_H_) / (MD_I_ + MD_H_). This MD-index was already used in a previous work for characterizing microstructural integrity within the salvaged penumbra [9].

The assessment of contralateral voxels was achieved by swapping the penumbral mask (without applied FAST, grey matter identification was made for the contralateral tissue separately within this procedure) into the contralateral hemisphere. For this purpose, penumbral masks must be aligned to MNI space by using diffusion-to-standard space transformations and their corresponding inverses. They were used to warp penumbral masks into MNI space, then swap them to the contralateral hemisphere (using FSL related tools) and align them back (over a step of grey matter identification (using FAST) in individual T1 space) to diffusion space where MD-value extraction was performed. For registering T1 images to the MNI ICBM 152 non-linear (6th Generation) symmetric standard-space T1-weighted average structural template image [10], a two-step transformation procedure was applied: In the first step, linear (affine) transformation was performed using FLIRT, followed by the second step, that used the generated output to perform non-linear registration with FNIRT (FMRIB's Non-Linear Image Registration Tool). The output of this transformation procedure was an individual native-to-standard (MNI space) non-linear warp field. The above-mentioned individual diffusion-to-native transformation matrix was combined with the just described native-to-standard non-linear transformation matrix, that resulted in diffusion-to-standard space transformations, that were used for alignment.

1 Straka M, Albers GW, Bammer R (2010) Real-time diffusion-perfusion mismatch analysis in acute stroke. J Magn Reson Imaging 32:1024-1037

2 Dehkharghani S, Bammer R, Straka M et al (2015) Performance and Predictive Value of a User-Independent Platform for CT Perfusion Analysis: Threshold-Derived Automated Systems Outperform Examiner-Driven Approaches in Outcome Prediction of Acute Ischemic Stroke. AJNR Am J Neuroradiol 36:1419-1425

3 Lansberg MG, Lee J, Christensen S et al (2011) RAPID automated patient selection for reperfusion therapy: a pooled analysis of the Echoplanar Imaging Thrombolytic Evaluation Trial (EPITHET) and the Diffusion and Perfusion Imaging Evaluation for Understanding Stroke Evolution (DEFUSE) Study. Stroke 42:1608-1614

4 Yushkevich PA, Piven J, Hazlett HC et al (2006) User-guided 3D active contour segmentation of anatomical structures: significantly improved efficiency and reliability. Neuroimage 31:1116-1128

5 Smith SM (2002) Fast robust automated brain extraction. Hum Brain Mapp 17:143-155

6 Zhang Y, Brady M, Smith S (2001) Segmentation of brain MR images through a hidden Markov random field model and the expectation-maximization algorithm. IEEE Trans Med Imaging 20:45-57

7 Jenkinson M, Smith S (2001) A global optimisation method for robust affine registration of brain images. Med Image Anal 5:143-156

8 Jenkinson M, Bannister P, Brady M, Smith S (2002) Improved optimization for the robust and accurate linear registration and motion correction of brain images. Neuroimage 17:825-841

9 Berndt MT, Maegerlein C, Boeckh-Behrens T et al (2020) Microstructural Integrity of Salvaged Penumbra after Mechanical Thrombectomy. AJNR Am J Neuroradiol 41:79-85

10 Grabner G, Janke AL, Budge MM, Smith D, Pruessner J, Collins DL (2006) Symmetric atlasing and model based segmentation: an application to the hippocampus in older adults. Med Image Comput Comput Assist Interv 9:58-66
